# Systematic review fracture resistance of endodontically treated posterior teeth restored with fiber reinforced composites- a systematic review

**DOI:** 10.1186/s12903-023-03217-2

**Published:** 2023-08-13

**Authors:** Harish Selvaraj, Jogikalmat Krithikadatta, Deepti Shrivastava, Meshal Aber Al Onazi, Hmoud Ali Algarni, Swapna Munaga, May Osman Hamza, Turki saad Al-fridy, Kavalipurapu Venkata Teja, Krishnamachari Janani, Mohammad Khursheed Alam, Kumar Chandan Srivastava

**Affiliations:** 1 Department of Conservative Dentistry and Endodontics, Saveetha Dental College and Hospitals, Saveetha Institute of Medical and Technical Sciences, Chennai, Tamil Nadu 152006005 India; 2 Department of Cariology, Saveetha Dental College & Hospitals, SIMATS, Chennai, India; 3 Department of Preventive Dentistry, College of Dentistry, Jouf University, Sakaka, Saudi Arabia; 4 Department of Operative Dentistry and Endodontics, College of Dentistry, Jouf University, Sakaka, 72345 Saudi Arabia; 5 Restorative and Prosthetic Dental Sciences, College of Dentistry, King Saud Bin, Abdulaziz University for Health Sciences, Riyadh, Saudi Arabia; 6 King Abdullah International Medical Research Center, Riyadh, Saudi Arabia; 7 Department of Prosthetic Dental Sciences, College of Dentistry, Jouf University, Sakaka, Saudi Arabia; 8 General Dentist, Ministry of Health, Riyadh, Saudi Arabia; 9 Department of Conservative Dentistry and Endodontics, Mamata Institute of Dental Sciences, Mamata Educational Society, Bachupally, Hyderabad 500 090 India; 10 Department of Conservative Dentistry and Endodontics, SRM Dental College, SRM Institute of Science & Technology, Chennai, India; 11 Department of Dental Research Cell, Saveetha Institute of Medical and Technical Sciences, Saveetha Dental College and Hospitals, Chennai, 600077 India; 12 Department of Public Health, Faculty of Allied Health Sciences, Daffodil International University, Dhaka, 1207 Bangladesh; 13 Department of Oral & Maxillofacial Surgery & Diagnostic Sciences, College of Dentistry, Jouf University, Sakaka, Saudi Arabia; 14 Department of Oral Medicine and Radiology, Saveetha Dental College, Saveetha Institute of Medical and Technical Sciences, Saveetha University, Chennai, 602105 India

**Keywords:** Endodontics, Post-endodontic dental restoration, Fiber-reinforced composites, Fracture strength, Polymeric composite biomaterials, Short e-glass fiber

## Abstract

**Background:**

Large cavity designs and access cavities impair endodontically treated tooth fracture resistance. As the tooth’s strength is known to reduce significantly after the root canal treatment, occlusal loading as a result of functions such as chewing, biting and certain parafunctional tendencies makes the endodontically treated tooth vulnerable to fracture. Hence, after endodontic treatment, it is vital to give adequate and appropriate restorative material to avoid tooth fractures. Accordingly, the choice of such restorative material should be dictated by the property of fracture resistance.

**Objective:**

The goal of this study was to conduct a systematic review and critical analysis of available data from in vitro studies examining the fracture resistance of endodontically treated posterior teeth restored with fiber-reinforced composites.

**Methodology:**

The Preferred Reporting Items for Systematic Review and Meta-Analysis (PRIS-MA) Statement was used to guide the reporting of this systematic review A comprehensive literature search was performed using MEDLINE (via PubMed), Scopus, ScienceDirect, Google Scholar, and LILACS. A manual search of the reference lists of the articles was also performed. The databases provided a total of 796 studies from the electronic systematic search. The databases provided a total of 796 studies from the electronic systematic search. Two reviewers scrutinized the papers for eligibility based on inclusion/exclusion criteria and extracted data. The studies were assessed for their potential risk of bias. Based on modified JBI & CRIS (checklist for reporting in vitro studies) guidelines, along with the methodology and treatment objective, we have formulated 13 parameters specifically to assess the risk of bias. A total of 18 studies met the inclusion criteria and were included for qualitative analysis. Considering the high heterogeneity of the studies included, a meta-analysis could not be performed.

**Results:**

The majority of the included studies had a moderate or high risk of bias. When compared to traditional hybrid composites, fiber-reinforced composites showed increased fracture resistance of endodontically treated teeth in the majority of investigations. On the other hand, limited evidence was found for the bulk fill composites. Moreover, moderate evidence was found for the fracture resistance of inlays and fiber posts with fiber-reinforced composites for core build-up in endodontically treated teeth. No evidence could be found comparing the fracture resistance of endo crowns and fiber-reinforced composites in endodontically treated teeth.

**Conclusion:**

According to the research, using fiber-reinforced composites instead of conventional hybrid composites improves the fracture resistance of endodontically treated teeth. However, there was a high risk of bias in the research considered. No judgments could be reached about the superiority of one material over another based-on comparisons between other core restorations.

## Introduction

Root canal treated teeth are more likely to fracture, resulting in a decrease in the resistance and fracture toughness. Physical characteristics such as tooth structure loss, cusps, ridges, and the arching roof of the pulp chamber contribute to this [1]. Structure loss is caused by caries, access cavity preparation, trauma and radicular preparation. The effects of chemicals and intracanal medicaments, influence the fracture resistance of endodontically treated teeth [2]. Endodontic access cavity preparations increased cuspal deflection and increased the risk of cusp breakage during function [3, 4]. Proprioception is impaired in root canal treated teeth [1, 5]. The survival of root canal treated teeth is determined by the efficacy of root canal therapy, as well as the amount of surviving dentine thickness and post-endodontic healing [6]. Only after an adequate permanent coronal restoration has been placed should the root canal procedure be considered complete. In endodontic clinical practice, the quality of the final restoration is critical as it reduces the microleakage [6].

With advancements in both fillers and polymer processes, newer composite materials now offer a wide range of qualities to meet the needs of each individual clinical circumstance [6, 7]. The use of an optimum material with adequate fracture resistance when restoring endodontically treated teeth is an essential aspect to consider during post-endodontic rehabilitation. Newer fiber-reinforced composite materials reinforce weaker tooth structurally and chemically. Fiber-reinforced composite can help prevent endodontically treated teeth from fracturing [8]. Because of their improved physical and mechanical qualities, fiber-reinforced composites have been advocated for the biomimetic replacement of dentine in wider cavities and endodontically treated teeth. It promotes mechanical retention, prevents fracture propagation, and provides strong chemical bonding between glass fibers and the resin matrix.

Ribbond is a reinforced ribbon with a high elastic modulus constructed of ultra-high molecular weight polyethylene fiber. To improve adherence to synthetic restorative materials, it is treated with cold gas plasma. The material’s fiber network allows forces to be transferred. When polyethylene and glass fibers are employed in composite resins, they operate as a stress reliever [9] and have higher fracture resistance and flexural modulus [10, 11].

EverX posterior is a combination of e-glass type of fillers and glass filler with barium. This type of composite manufacturer affirms that the short-fiber composites strengthened the restoration by reducing the incidence of fracture, which leads to post-endodontic restoration failure. In vitro research showed that these two materials improved the resistance to fracture. There are a few unsolved problems about the fracture resistance of fiber-reinforced composites, such as whether they are more resistant to fracture than traditional microhybrid, nanohybrid composites, and other in-direct restorations?

We conducted a systematic review of published in vitro studies comparing the fracture resistance of fiber-reinforced composites with different restorations (hybrid composites, fiber posts, ceramic inlays, lithium disilicate endocrowns, and crowns) in endodontically treated teeth due to a lack of sufficient evidence. Therefore, the goal of this study is to compare the fracture resistance of endodontically treated teeth repaired using fiber-reinforced composites to that of other core restorations in vitro tests. The research question was: are fiber-reinforced composites more resistant to fracture than other core restorations in endodontically treated teeth? The null hypothesis states that fiber-reinforced composites are less resistant to fracture than conventional microhybrid and nanohybrid composites, fiber-reinforced posts, crowns (with or without posts), lithium disilicate endocrowns and ceramic inlay in endodontically treated teeth. Whereas the alternate hypothesis states fiber-reinforced composites are more resistant to fracture than conventional microhybrid and nanohybrid composites, fiber-reinforced posts, crowns (with or without posts), lithium disilicate endocrowns and ceramic inlay in endodontically treated teeth.

## Materials and methods

The study protocol was registered on the PROSPERO database (http://www.crd.york.ac.uk) under number CRD42021295212 on 30/12/2021. The Preferred Reporting Items for Systematic Review and Meta-Analysis (PRISMA) Statement was used to guide the reporting of this systematic review.Study Design/Study Setting- Only in vitro studies were considered for this review

### The data sources and the literature search strategy

To find publications published in English only, a full electronic exploration was conducted in MEDLINE (via PubMed), Scopus, ScienceDirect, Google Scholar, and LILACS. The research question was written in its free form as follows: In endodontically treated teeth, are fiber-reinforced composites more resistant to fracture than alternative core restorations? For the structured review question, the PICOS (population, intervention, comparison, and outcome) technique was used

- Population - Fully formed extracted human teeth which are endodontically treated.
- Intervention - Dental restorative Fiber-reinforced Composites.
- Comparison - Conventional hybrid and nanohybrid composites, fiber-reinforced posts, crowns (with or without posts), endocrowns, ceramic inlay.
- Outcome - Evaluation of fracture resistance using a universal testing machine.

The published research papers between January 2000 and May 2023 were reviewed. The search terms were as follows: fiber-reinforced composites, short fiber composite, EverXposterior, Ribbond, fracture resistance and fracture strength. These keywords were combined as ((((((((((((Short fiber-reinforced composite) OR (short fiber-reinforced composite)) OR (fiber-reinforced composite)) OR (short fiber composite)) OR (e-glass fiber)) OR (Fiber-reinforced composites)) OR (EverX Posterior)) OR (Rib-bond)) OR (polyethylene fiber ribbon)) AND ((((((((((((nanocomposite) OR (nanofilled composite)) OR (nano hybrid composite)) OR (micro filled composite)) OR (microhybrid composite)) OR (fiber post)) OR (Fiber-reinforced composite post)) OR (Inlay)) OR (onlay)) OR (crowns)) OR (endo crowns)) OR (indirect restorations))) AND ((((endodontically treated teeth) OR (structurally compromised teeth)) OR (traditional access cavity)) OR (conventional access cavity))) AND (((((((Fracture resistance) OR (Flexural strength)) OR (Fracture toughness)) OR (Modulus of Rupture)) OR (Flexural Resistance)) OR (Fracture Strength)) OR (Bend Strength))). These terms and keywords were taken from published research papers in the journals: Journal of Endodontics, International Endodontic Journal, and Australian Endodontic Journal. Each database’s search terms were changed. Added research articles were not identified through the previous approaches, but were hand-searched in the reference lists of all included articles (Table 1a and 1b).

**Table 1a:** Results of PUBMED bibliometric search engines between 2010–2023

Search Number	Query	Results	Time
5	(((((((((((Short fiber-reinforced composite) OR (short fiber-reinforced composite)) OR (fiber-reinforced composite)) OR (short fiber composite)) OR (e-glass fiber)) OR (Fiber-reinforced composites)) OR (EverX Posterior)) OR (Ribbond)) OR (polyethylene fiber ribbon)) AND ((((((((((((nanocomposite) OR (nanofilled composite)) OR (nano hybrid composite)) OR (micro filled composite)) OR (microhybrid composite)) OR (fiber post)) OR (Fiber-reinforced composite post)) OR (Inlay)) OR (onlay)) OR (crowns)) OR (endo crowns)) OR (indirect restorations))) AND ((((endodontically treated teeth) OR (structurally compromised teeth)) OR (traditional access cavity)) OR (conventional access cavity))) AND (((((((Fracture resistance) OR (Flexural strength)) OR (Fracture toughness)) OR (Modulus of Rupture)) OR (Flexural Resistance)) OR (Fracture Strength)) OR (Bend Strength))	152	03:20:58
4	((((((Fracture resistance) OR (Flexural strength)) OR (Fracture toughness)) OR (Modulus of Rupture)) OR (Flexural Resistance)) OR (Fracture Strength)) OR (Bend Strength)	36,854	03:20:41
3	(((endodontically treated teeth) OR (structurally compromised teeth)) OR (traditional access cavity)) OR (conventional access cavity)	5,887	03:20:25
2	(((((((((((nanocomposite) OR (nanofilled composite)) OR (nano hybrid composite)) OR (micro filled composite)) OR (microhybrid composite)) OR (fiber post)) OR (Fiber-reinforced composite post)) OR (Inlay)) OR (onlay)) OR (crowns)) OR (endo crowns)) OR (indirect restorations)	134,928	03:20:05
1	((((((((Short fiber-reinforced composite) OR (short fiber-reinforced composite)) OR (fiber-reinforced composite)) OR (short fiber composite)) OR (e-glass fiber)) OR (Fiber-reinforced composites)) OR (EverX Posterior)) OR (Ribbond)) OR (polyethylene fiber ribbon)	5,803	03:19:39

**Table 1b:** Results of other bibliometric search databases between 2010–2023

Database Searched	Query	No. of Search Results
ScienceDirect	(Fiber reinforced composites) AND (direct composite restorations or indirect restorations) AND (endodontically treated teeth) AND (Fracture resistance OR Fracture strength or fracture toughness); Year(s): 2010–2023	148
LILACS	Endodontically treated teeth [Words] and Fiber reinforced composite [Words] and Fracture resistance [Words]	0
Google Scholar	“fiber reinforced composite” AND “direct composite restoration” OR “indirect restoration” AND “endodontically treated teeth” AND “Fracture resistance” OR “fracture strength” OR “fracture toughness” Year(s) : 2010–2023	279
Scopus	“fiber reinforced composite” AND “direct composite restoration” OR “indirect restoration” AND “endodontically treated teeth” AND “Fracture resistance” OR “fracture strength” OR “fracture toughness” Year(s) : 2010–2023	217

### Screening and selection of the studies

Two independent reviewers assessed whether the title of the article identified through the electronic database was appropriate with the review question under the guidance of an expert third reviewer. After then, the abstracts were rigorously scrutinized in order to identify research that was eligible. If the information gathered from the title and abstract was not sufficient, the full text of the article was examined. In the event of disagreement between the reviewers on inclusion or exclusion of studies, a third reviewer was involved to achieve consensus. Only studies that matched all of the following criteria were considered for inclusion:

- In vitro studies assessing the fracture resistance of fiber-reinforced composites in Endodontically treated teeth.
- In vitro studies assessing the fracture resistance of fiber-reinforced composites in Endodontically treated posterior teeth.
- Studies which assessed the fracture resistance of fiber-reinforced composites in different cavity configurations.
- Studies comparing the resistance to fracture of root canal treated teeth treated with fiber-reinforced composites to conventional hybrid composites, inlays, crowns, fiber posts, and endocrowns.

Exclusion Criteria

- Animal studies and case reports.
- Studies that have used other material such as EverStick.
- Studies assessing fracture resistance of fiber-reinforced composites in teeth without endodontic therapy.
- Studies done in anterior teeth.

### Assessment of risk of bias (ROB)

Since there is no clearly defined risk of bias tool to assess in vitro studies, based on modified JBI & CRIS (checklist for reporting in vitro studies) guidelines, along with the methodology and treatment objective [12], we have formulated 13 parameters specifically to study the fracture resistance. Randomization, use of control standard, standardization of teeth, age, method of sample size estimation, material based on manufacturer’s instructions, samples prepared by a single operator, observer blinding, thermocycling, cyclic loading, periodontal ligament simulation, mode of fracture were examined. The article will be marked a “Yes” on that parameter if the authors reported it; if the information could not be retrieved, then it’s reported as “No.“

Each article was evaluated by the means of Risk of Bias score. The important parameters for fracture resistance studies such as standardization of teeth dimensions, usage materials as directed by the manufacturer, thermocycling, cyclic loading, axial loading direction, periodontal ligament simulation and mode of fracture were given higher weightage and a score of “2” and other parameters were given a score of “1” if the articles recorded a “Yes” in these parameters. The articles were assessed to have a “High” if the ROB score was less than 10, “Moderate” if ROB score is between 10–14, and “Low” if ROB score was more than 14. The two reviewers made their assessments in-dependently, with any disputes addressed by consensus. Every attempt was undertaken to get any missing information from the listed research. Missing information was sought by sending emails to the authors of the papers listed.

### Data extraction

All relevant papers’ full texts were retrieved, and the data was extracted simultaneously by two reviewers using a consistent outline. Authors names, published year, type of teeth, details of control groups, cavity configuration, techniques used for root canal preparation, apical diameter, disinfecting agents, method of canal obturation, sealer used, materials evaluated, material used for fracture testing and crosshead speed, interpretation of results (N, kg, or lb), and assessment of outcomes. The information was gathered from the tests to see how different fiber-reinforced composites and specific fibers affected the resistance to fracture. Every included paper was examined for commonalities in order to conduct a meta-analysis. A meta-analysis, however, was not possible due to the heterogeneity of the studies. The findings of investigations on the impact of fiber-reinforced composites on tooth fracture resistance were compiled. The following is an example of evidence synthesis: [12]

1. Strong evidence: information from two or more high-quality studies with usually consistent findings across all investigations (≥ 75%of studies found consistent findings).
2. Moderate evidence: 1 high-quality study and/or 2 or more low-quality studies with generally consistent findings across all investigations (≥ 75%of studies reported consistent findings).
3. Limited evidence: based on only one low-quality study.
4. Contradictory evidence: Inconsistent outcomes across several studies (< 75% of studies reported consistent results).
5. No evidence: There were no studies found.

## Results

### Search results

**Fig. 1:**
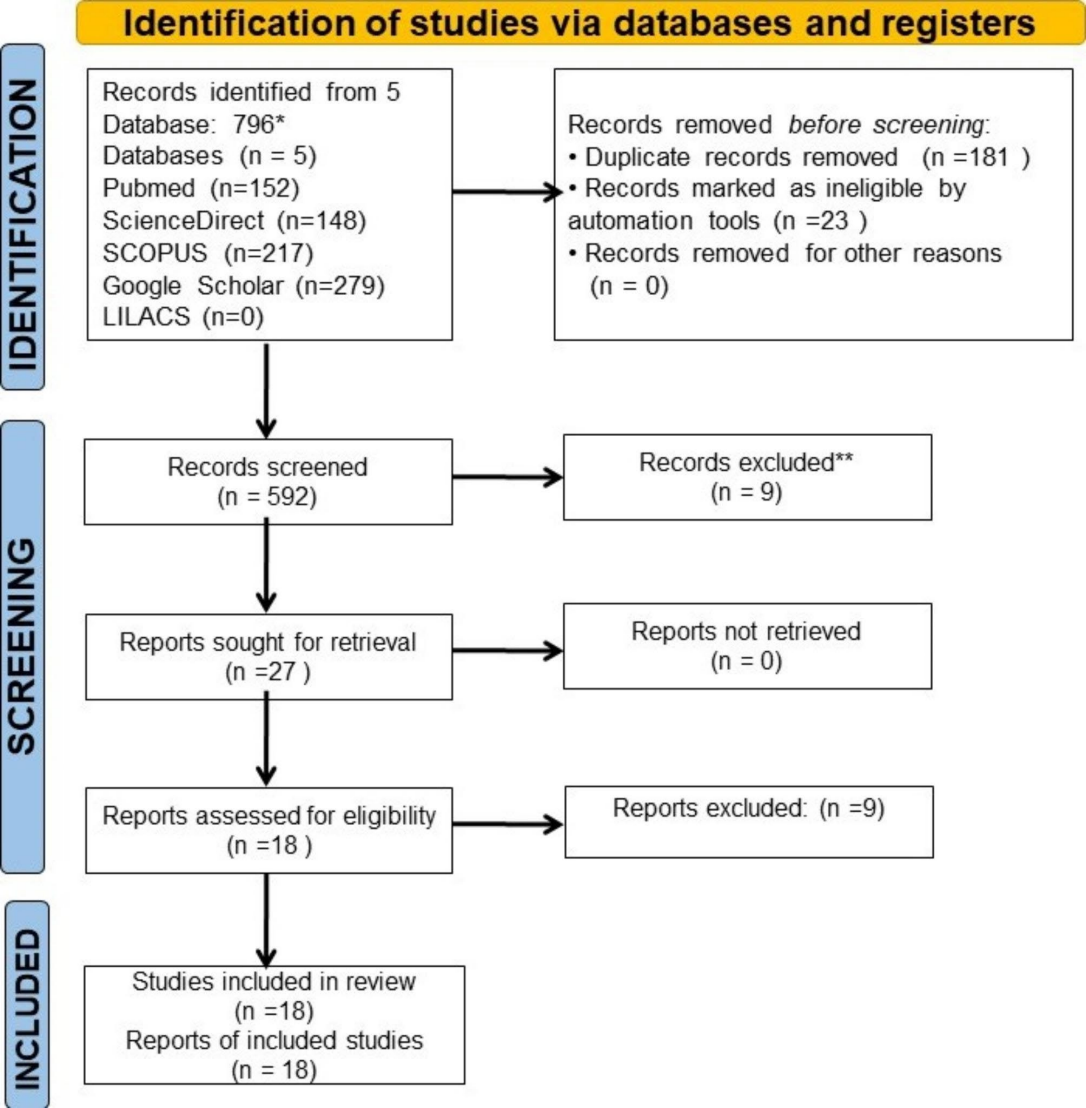
Flowchart of the systematic review process *Consider, if feasible to do so, reporting the number of records identified from each database or register searched (rather than the total number across all databases/registers). **If automation tools were used, indicate how many records were excluded by a human and how many were excluded by automation tools.

The databases provided a total of 796 studies from the electronic systematic search. PubMed identified 152 records, Scopus identified 217 records, ScienceDirect identified 148 items, and Google Scholar identified 279 records. The duplicates were removed using the Rayyan AI tool. After the removal of duplicates and data screening based on the title and abstract, 25 articles were selected for full-text reading. (Fig. 1). A total of 9 articles [13–17]2 [18] were eliminated after full text reading and the reason for exclusion has been discussed in Table 2. After full-text reading, 18 papers [19–35] were identified as being eligible for this systematic review (Table 3).

**Table 2:** Characteristics of excluded articles

S.No	Author	Year	Reason for exclusion
1.	Aslan. et al. [36]	2017	Polyethylene fiber ribbon used only in the occlusal surface.
2.	Rocca. et al. [15]	2015	The crowns of all teeth were cut 2 mm above CEJ without any specific cavity configuration.
3.	Scotti. et al. [16]	2015	Only a single layer of individual fibers placed in a single direction.
4.	Basaran. et al. [17]	2019	Fiber only on the cavity floor.
5.	de Kuijper. et al. [19]	2019	Entire crown structure removed.
6.	Patnana et al. [18]	2020	Study was done in anterior teeth with simulated incisal fractures.
7.	Kassis. et al. [37]	2021	Use of fiber reinforced composite as a substrate for dentin replacement before inlay or onlay preparation and no comparison to direct composite restoration with fiber reinforced composites.
8.	Frater. et al. [38]	2021	No endodontic therapy carried out in samples.
9.	Sharma .et al [39]	2022	Comparison of Endodontically treated teeth restored with Filtek P60 composite to radicular posts with EverX flow at different depths.

**Table 3:** Descriptive data of included studies

Author and Year of publication	Study Title	Type of Teeth	Presence of Control Groups	Cavity Configuration	Cleaning and shaping	Obturation	Mechanical testing	Materials Evaluated	Type of Fracture	Interpretation
Cobankara et al. 2008 [40]	The Effect of Different Restoration Techniques on the Fracture Resistance of Endodontically-treated Molars	Mandibular Molars	PC, NC	MOD	Step back technique with hand instruments upto #35. Irrigation performed using 5.25% NaOCl	AH Plus, CLC	Cross head speed 1 mm/minute, 6 mm stainless steel bar used to fracture	1. CavexAvalloy-II spill, Lathe-cut silver alloy for dental amalgam 2. ClearfilPhotoposterior, Kuraray 3. Estenia Indirect Hybrid Ceramic Inlay 4. Polyethylene Ribbon Fiber (Ribbond)	Favourable	Indirect hybrid ceramic inlay seemed more reliable because of higher fracture strength and prevention of unfavourable fractures.
Srirekha et al. 2012 [21]	The reinforcement effect of polyethylene fiber and composite impregnated glass fiber on fracture resistance of endodontically treated teeth	Maxillary Premolars	PC	MOD	Rotary instrumentation with ProTaper Files upto F2. Irrigation performed using 5.25% NaOCl	AH Plus, CLC,SC	NM, 5 mm diameter round stainless steel ball used to fracture	1. Filtek Z350 XT 2. Interlig; Angelus 3. Ribbond	NM	Composite impregnated glass fiber-reinforced group possessed higher Fracture Strength.
Khan et al., 2013 [41]	Effect of Two Different Types of Fibers on the Fracture Resistance of Endodontically Treated Molars Restored with Composite Resin	Mandibular Molars	PC, NC	MOD	Step back technique with hand instruments upto #35 for Distal canals and #40 for mesial canals. Irrigation performed using 5.25% NaOCl	AH Plus, CLC	0.5 mm/min, 6 mm diameter stainless steel bar used to fracture	1. Hybrid Resin Composite, Venus Heraeus Kulzer 2. Leno Woven Ultrahigh Molecular weight (LWUHM) polyethylene fiber (Ribbond; Seattle, WA, USA) 3.Vectris®Ivoclar Vivadent	NM	Insertion of polyethylene ribbon fibers in root filled molars with MOD preparation significantly increased the fracture strength.
Kalburge et al., 2013 [23]	A comparative evaluation of fracture resistance of endodontically treated teeth, with variable marginal ridge thicknesses, restored with composite resin and composite resin reinforced with Ribbond: An in vitro study	Maxillary Premolars	PC, NC	MOD	NM	NM	2 mm/min	1. Filtek Z‑100 (3 M ESPE) 2. Polyethylene Fiber Ribbond (Ribbond; Seattle, WA, USA)	NM	On static loading, preserving the mesial marginal ridge of Composite‑restored and Ribbond‑reinforced composite‑restored maxillary premolars can help preserve the fracture resistance of teeth.
Costa et al., 2014 [42]	Fracture resistance of mechanically compromised premolars restored with polyethylene fiber and adhesive materials	Maxillary Premolars	PC	MODP	Rotary instrumentation with ProTaper Files upto F3. Irrigation performed using 1% NaOCl	AH Plus, CLC,SC	1 mm/min, Rectangular round-tipped metal point used to fracture	1. Filtek Z250;3 M ESPE 2. Fiber Post(Angelus,Londrina) 3. Ribbond (Ribbond; Seattle, WA, USA)	Favourable	Ribbon–fiber-reinforced resin restorations provided superior fracture resistance of premolars with MODP and endodontic access cavities.
Kemaloglu et al. 2015 [43]	Effect of novel restoration techniques on the fracture resistance of teeth treated endodontically: An in vitro study	Mandibular Premolars	No Control Group	MOD	Rotary instrumentation with ProTaper Files upto F5. Irrigation performed using 2.5% NaOCl	AH Plus, SC	1 mm/min, Modified steel Ball used to fracture	1. Filtek Z550;3 M ESPE 2. Ribbond (Ribbond; Seattle, WA, USA) 3. EverX Posterior (GC everX posterior, GC Corp) 4. Filtek Bulk Fill;3 M ESPE	Favourable	Fiber-reinforcement improved the fracture strength of teeth with large MOD cavities treated endodontically.
Atalay et al. 2016 [26]	Fracture Resistance of Endodontically Treated Teeth Restored With Bulk Fill, Bulk Fill Flowable, Fiber-reinforced, and Conventional Resin Composite	Maxillary Premolars	PC, NC	MOD	Rotary instrumentation with ProTaper Files upto F3. Irrigation performed using 5.25% NaOCl	AH Plus, SC	1 mm/min, Steel sphere 8 mm in diameter used to fracture	1. Filtek Bulk Fill Posterior Restorative 2. Bulk Fill Flowable Composite (SureFil SDR Flow) 3. Fiber-reinforced composite (GC everX posterior, GC Corp) 4. Conventional Nanohybrid Resin Composite Tetric N-Ceram, (Ivoclar/Vivadent)	Favourable	The fracture resistance values of endodontically treated teeth restored with either bulk fill/bulk fill flowable or fiber-reinforced composite were not different from those restored with conventional nanohybrid resin composite.
Bilgi et al. 2016 [44]	Comparison of fracture resistance of endodontically treated teeth restored with nanohybrid, silorane, and fiber-reinforced composite: An in vitro study	Maxillary Premolars	PC	MOD	Step back technique with hand instruments. Irrigation performed using 5.25% NaOClv	AH Plus, CLC	1 mm/min, 0.5 mm diameter round bar used to fracture	1. Conventional nanohybrid composite + Glass fiber 2. Silorane Composites 3. EverXposterior (GC everX posterior, GC Corp)	NM	Among the experimental groups, fiber‑reinforced composite showed the highest fracture resistance.
Gürel et al. 2016 [45]	Fracture Resistance of Premolars Restored Either with Short Fiber or Polyethylene Woven Fiber-Reinforced Composite	Maxillary Premolars	No Control Group	MODP	Rotary instrumentation with ProTaper Files upto F5. Irrigation performed using 2.5% NaOCl	AH Plus, SC	1 mm/min, Stainless steel ball 4 mm in diameter used to fracture	1. SFRC (EverX Posterior, GC) 2. Conventional Filler Composite (G-aenial Posterior, GC) 3. PWFP post (Ribbond thin, Ribbond Inc; Seattle, WA)	Favourable	The restoration of severely weakened premolar teeth with the use of short fiber-reinforced composite might have advantages over conventional filler composite or polyethylene woven fiber-reinforced composite techniques.
Yasa et al. 2016 [46]	Effect of novel restorative materials and retention slots on fracture resistance of endodontically-treated teeth	Mandibular Molars	PC	MOD	Rotary instrumentation with ProTaper Files upto F2 in Mesial Canals, F3 in Distal Canals. Irrigation performed using 2.5% NaOCl, 17% EDTA	AH Plus, SC	1 mm/min, Steel spherical tip with a diameter of 6 mm used to fracture	1. Nano-hybrid composite resin (FiltekTM Z550 ;3 M ESPE) 2. Bulk-fill flowable (FiltekTM Bulk Fill ;3 M ESPE) 3. Short fiber-reinforced-composite (everX Posterior TM)	Non Favourable	The use of short fiber-reinforced composite with retentive slots could prevent cuspal fracture on endodontically-treated teeth with MOD cavity.
Ozsevik et al. 2016 [30]	Effect of fiber-reinforced composite on the fracture resistance of endodontically treated teeth	Mandibular Molars	PC, NC	MOD	Step back technique with hand instruments upto #35. Irrigation performed using NaOCl	AD Seal (MetaBiomed, CLC	1 mm/min, Steel round-shaped tip with a diameter of 5 mm used to fracture	1. G–ænial posterior, GC Corporation 2. Ribbond (Ribbond Inc; Seattle, WA) 3. EverXposterior, GC Corporation	NM	EverX posterior under composite restorations resulted in fracture resistance similar to that of intact teeth.It reinforced root-filled teeth more than composite alone and ribbon and composite restorations.
Eapen et al. 2017 [47]	Fracture Resistance of Endodontically Treated Teeth Restored with 2 Different Fiber-reinforced Composite and 2 Conventional Composite Resin Core Buildup Materials	Maxillary Premolars	PC, NC	MOD	Step back technique with hand instruments upto #40. Irrigation performed using 5.25% NaOCl	AH Plus, CLC	1 mm/min, Metal indenter with a 6-mm diameter used to fracture	1. Dual Cure Composite MutiCore Flow (Ivoclar Vivadent) 2. Posterior Resin Composite Filtek P60 (3 M ESPE) 3. Fiber-reinforced Composites- Interlig Fiber (Angelus) 4. Short Fiber Composites EverXPosterior (GC Company)	Favourable	Short fiber-reinforced composite can be used as a direct core buildup material that can effectively resist heavy occlusal forces against fracture and may reinforce the remaining tooth structure in endodontically treated teeth.
T.G. Garlapati et al. 2017 [48]	Fracture resistance of endodontically treated teeth restored with short fiber composite used as a core material	Mandibular Molars	PC, NC	MOD	Step back technique with hand instruments upto #35 for Distal canals and #30 for Mesial Canals. Irrigation performed using 3% NaOCl	AH Plus, CLC	0.5 mm/min, 6 mm stainless steel sphere used to fracture	1. Hybrid composite (Te-EconomPlus,IvoclarVivadent, Asia) 2. Leno Woven Ultrahigh Molecular weight (LWUHM) polyethylene fiber(Ribbond; Seattle, WA, USA) 3. everX posterior (GC EUROPE)	Favourable	Endodontically treated teeth restored with EverX posterior fiber-reinforced composite showed superior fracture resistance.
Özyürek et al. 2018 [33]	The Effects of Endodontic Access Cavity Preparation Design on the Fracture Strength of Endodontically Treated Teeth: Traditional Versus Conservative Preparation	Mandibular Molars	PC	Conservative Endodontic Access and Traditional Access	Rotary instrumentation with ProTaper Next Files upto X2 for Mesial Canals, X4 for Distal Canals. Irrigation performed using 5.25% NaOCl	AH Plus, SC	1 mm/min, 6-mm round-head tip used to fracture	1. EverX posterior (GC EUROPE) 2. SDR (Dentsply Caulk, Milford, DE)	Favourable in ExP in CEC and Unfavorable in ExP in TEC	The fracture strength of teeth restored with the SDR bulk-fill composite was higher than that of teeth restored with EverX Posterior.
Shah et al. 2020 [34]	Performance of fiber-reinforced composite as a post-endodontic restoration on different endodontic cavity designs	Maxillary Premolars	PC, NC	MO & MOD	Step back technique with hand instruments upto #35. Irrigation performed using 5.25% NaOCl	AH Plus, CLC	0.5 mm/min, 6 mm Stainless steel sphere used to fracture	1. Hybrid composite (Te-Econom Plus, Ivoclar Vivadent, Asia) 2. EverX Posterior GC Corporation,Europe 3. Leno Woven Ultrahigh Molecular weight (LWUHM) polyethylene fiber (Ribbond; Seattle, WA, USA)	Favourable	Fiber-reinforced composites when used in different cavity configurations of endodontically treated Premolar yielded similar results. More favourable fractures were seen in teeth restored with fiber-reinforced composites when compared to conventional composites
Donova et al. 2019 [49]	Direct bilayered biomimetic composite restoration: The effect of a cusp-supporting short fiber-reinforced base design on the chewing fracture resistance and failure mode of molars with or without endodontic treatment	Maxillary Third Molars	PC	MODP	Protaper upto F3, 2.5% Sodium Hypochlorite used as an irrigant	AH Plus and CLC	Cross head speed of 1 mm/min. Metal sphere with a diameter of 6 mm with tripod contact (the mesiobuccal, distobuccal and mesiopalatal cusps)	Direct composite resin (GC Posterior, GC, Tokyo, Japan) and Short-FRC (everX Posterior, GC Corporation, Tokyo, Japan)	Cavities restored without SFRC base showed unfavourable mode of fracture	A cusp-supporting design made of a short-FRC base (everX Posterior) improved the chewing fracture resistance and fracture manner of compromised molars.
Frankenberger et al. 2021 [38]	Post-Fatigue Fracture and Marginal Behavior of Endodontically Treated Teeth: Partial Crown vs. Full Crown vs. Endocrown vs. Fiber-Reinforced Resin Composite	Mandibular 3rd Molars	PC	MOD	MTwo upto size 0.04/#40, Irrigation solutions not mentioned.	AH Plus and CLC	0.5 mm/min. Steatite ball, 6 mm diameter.	Tetric EvoCeram BulkFill bonded with AdheSE Universal, EverX Posterior bonded with G-Premio Bond, e.max CAD, Celtra Duo Partial and Full Crowns, Zirconia Partial and Full Crowns, indirect non-bonded cast gold restorations.	All the failed Restorations showed an Unfavourable mode of fracture	Indirect restoration with cuspal coverage is suitable for the restoration of endodontically treated teeth in MOD cavities. All indirect restorations showed a promising performance after in vitro fatigue-loading compared to direct composite and fiber reinforced composite.
Volom et al. 2023 [35]	Fatigue performance of endodontically treated molars reinforced with diferent fiber systems	Mandibular Molars	Control (MOD Cavities restored with FRCs)	MOD	ProTaper upto F3, Irrigation with 5% NaOCl and 10% EDTA	AH Plus and Matched Single Cone	Round-shaped metallic tip 6 mm in diameter, Cross head speed not mentioned	EverX Flow, EverX Flow + G-aenial Injectable, Ribbond + G-aenial Posterior, Ribbond + G-aenial Flow, Fiber Post (FibreKleer, Petron, Orange, CA, USA), Fiber Post (FibreKleer, Petron, Orange, CA, USA) + G-aenial Flow	NM	Teeth restored with Short Fiber reinforced Composite restorations performed better without Cuspal Coverage compared to the ones where Short Fiber reinforced Composite was covered. For MOD cavities in endodontically treated molars, direct cuspal coverage is recommended when utilizing long continuous fbers for reinforcement.

### Risk of bias

All the included studies were assessed for risk of bias. Of the 18 studies, 5 studies presented low risk of bias, 9 studies presented moderate risk of bias and 4 studies reported high ROB. The results are depicted in Table 4.

**Table 4:** Risk of bias of included studies

Author	Sample Size Calculation	Control Group	Cavity Dimensions	Age of patients	Teeth Randomization	Manufacturers Instruction	Single Operator	Blinding of the observer	Thermocycling	Cyclic Loading	Axial Loading Direction	Periodontal Ligament Simulation	Mode of Fracture	ROB Score	ROB
Cobankara et al. 2008 [40]	N	Y	Y	N	Y	Y	Y	N	Y	Y	Y	Y	Y	17	Low
Srirekha et al. 2012 [21]	N	Y	Y	N	Y	Y	N	N	Y	N	Y	Y	N	12	Moderate
Khan et al., 2013 [50]	N	Y	Y	N	Y	N	N	N	N	N	Y	N	N	6	High
Kalburge et al., 2013 [23]	N	Y	Y	N	Y	N	N	N	Y	N	Y	N	N	9	High
Costa et al., 2014 [51]	N	Y	Y	N	Y	Y	Y	N	Y	Y	N	Y	Y	15	Low
Kemaloglu et al. 2015 [52]	N	N	Y	N	Y	Y	N	N	Y	N	Y	Y	Y	13	Moderate
Atalay et al. 2016 [26]	N	Y	Y	N	Y	N	Y	N	Y	N	Y	Y	Y	13	Moderate
Bilgi et al. 2016 [44]	N	Y	Y	N	Y	Y	N	N	Y	N	N	Y	N	10	Moderate
Gürel et al. 2016 [45]	N	N	Y	N	Y	N	N	N	N	N	Y	N	Y	7	High
Yasa et al. 2016 [46]	N	Y	Y	N	N	Y	Y	N	N	N	N	N	Y	8	High
Ozsevik et al. 2016 [30]	Y	Y	Y	N	Y	Y	N	N	Y	N	Y	N	N	11	Moderate
Eapen et al. 2017 [53]	Y	Y	Y	N	Y	Y	N	N	N	N	Y	N	Y	11	Moderate
T.G. Garlapati et al. 2017 [48]	N	Y	Y	N	Y	Y	Y	N	Y	N	Y	Y	Y	15	Low
Özyürek et al. 2018 [33]	Y	Y	Y	N	Y	Y	N	N	N	N	Y	Y	Y	14	Moderate
Shah et al. 2020 [34]	N	Y	Y	N	Y	Y	N	N	Y	N	Y	Y	Y	14	Moderate
Donova et al. 2019 [49]	N	Y	Y	N	Y	Y	N	Y	Y	Y	Y	N	Y	17	Low
Frankenberger et al. 2021 [54]	Y	Y	N	N	Y	Y	N	Y	Y	Y	Y	N	Y	14	Moderate
Volom et al. 2023 [35]	Y	Y	Y	Y	N	Y	Y	Y	Y	N	N	Y	N	16	Low

### Cavity configuration

In the included studies, two studies had evaluated the fracture resistance of fiber-reinforced composites on Mesial-Occlusal Distal with palatal cusp removed (MODP) [24, 45]. The study by [33] have assessed the fracture resistance of fiber-reinforced composites in Traditional and Conservative access cavities of endodontically treated teeth. Rest all of the studies have assessed the fracture resistance of fiber composites in Class-II Mesial Occlusal Distal cavities of endodontically treated teeth. The study done by [33, 34] have evaluated fracture resistance in Class-II Mesial Occlusal cavities in addition. All studies have considered these cavity configurations to simulate heavily weakened teeth. Only one study [40], reported fiber-reinforced composites were unable to entirely restore the lost fracture resistance of MOD cavities which are endodontically treated. This study [33] reported no difference in the fracture resistance of Endodontically treated teeth restored with fiber-reinforced composites in Traditional and Conservative access cavities. While the remaining 14 studies reported that fiber-reinforced composites improved the fracture resistance of MOD and MODP access cavities of endodontically treated teeth [19–34].

### Type of teeth

The fracture resistance of fiber-reinforced composites in molars and premolars has been researched in the studies mentioned. Eight research [20, 22, 29, 33, 35, 49, 54, 55] assessed the fracture resistance of fiber-reinforced composites in endodontically treated molars, and ten looked at the fracture resistance of fiber-reinforced composites in endodontically treated premolars [21, 23–28, 30, 31, 34].The fracture resistance was greater in molars compared to premolars in Class II MOD access cavities of endodontically treated teeth as the volume of the remaining tooth structure was higher in molars.

### Mechanical testing

Universal testing machine. spherical stainless-steel ball compression loading was applied and fracture resistance testing was done based on static and dynamic loading amongst all included studies. Most of the included studies have performed the fracture testing using the cross-head speed of 1 mm/minute, whereas remaining studies have performed with either 0.5 mm/minute or 2 mm/minute. Also, the diameter of the steel ball varied from 4 to 8 mm.

### Fiber-reinforced composites vs. indirect core restorations

One study by [40] compared fracture resistance fiber-reinforced composites to Inlays in MOD access cavities of endodontically treated teeth reported that Inlays produced more favorable fractures which could be repaired if desired and may be recommended in restoring endodontically treated teeth indicating moderate evidence. One study by [54] assessed in vitro post-fatigue fracture behavior of endodontically treated molars with MOD cavities restored with fiber reinforced composites, partial and full crowns of e.max CAD, Celtra Duo, zirconia and cast gold restorations found that the indirect restorations of partial and full crowns should be considered than direct restorations with fiber reinforced composite or direct composite when restoring endodontically treated teeth with MOD cavities. The less invasive approach of direct restoration did not result in superior post-fatigue resistance but resulted in gap-free enamel margins compared to indirect restorations. Two studies assessed the use of fiber posts in endodontically treated teeth, of which one study [24] reported the use of fiber posts, polyethylene fibers and composite resin for core buildup resulted in higher fracture resistance of MODP access cavities of endodontically treated teeth and the study by [28] reported short fiber-reinforced composite (EverXposterior) possessed higher fracture strengths than Fiber-reinforced post (PWFP Ribbond) in MODP cavities of endodontically treated teeth. Therefore, the review found moderate evidence for the fracture resistance of fiber-reinforced posts with fiber-reinforced composites for core build-up compared to fiber-reinforced composites in MODP cavities of endodontically treated teeth.

### Fiber-reinforced composites vs. amalgam

Fiber-reinforced composites had higher fracture resistance than amalgam used for core material in MOD cavities of endodontically treated teeth, according to one study [40], with moderate evidence. Also, the use of amalgam resulted in fractures with root involvement (Unfavorable).

### Fiber-reinforced composites vs. bulk fill composites

In endodontically treated teeth, five papers investigated the fracture resistance of bulk fill composites with fiber-reinforced composites [20, 25, 26, 28, 33]. According to one study [26], there were no significant changes in fracture resistance values in endodontically treated teeth between conventional hybrid, bulk fill composites, and fiber-reinforced composites. SDR Bulk fill composite has higher resistance to fracture in root canal treated teeth than fiber-reinforced composites, according to one study ( [56–60]). Fiber-reinforced composites outperformed bulk fill composites in endodontically treated teeth, according to two investigations [20, 25, 26, 29]. As a result, the review found conflicting data concerning Bulk fill composites’ fracture resistance in endodontically treated teeth when compared to fiber-reinforced composites.

### Fiber-reinforced composites vs. conventional hybrid composites

Sixteen of the included studies have compared fiber-reinforced composites to conventional hybrid composites in endodontically treated teeth [35, 49, 59, 61–74]. Root canal-treated teeth treated with fiber-reinforced composites and hybrid composites showed no significant differences in fracture resistance in three studies [54, 58, 75, 76]. Fifteen studies [60, 77–90]; [35, 49, 54] reported consistent findings that fracture resistance of fiber-reinforced composites is higher when compared to conventional hybrid composites in endodontically treated teeth indicating strong evidence.

### Fiber-reinforced composites vs. silorane composites

The fracture resistance of endodontically treated teeth restored with fiber-reinforced composites was higher than that of silorane composites, according to one study [91], showing insufficient evidence.

### Type of fiber-reinforced composites

EverXposterior is a packable restorative fiber-reinforced composite whereas Ribbond, Vectris and Interlig are strips of fibers which are cut, placed and retained in the cavity using a stabilizing composite or a flowable composite incrementally. Thirteen studies reported the use of EverXposterior in endodontically treated teeth [25–35, 49, 54]. The study by [26] reported no significant difference between EverXposterior and conventional hybrid composites. Low viscosity bulk fill composites (SDR) had stronger fracture resistance in endodontically treated teeth than fiber-reinforced composites, according to one study [33]. EverXposterior enhanced the fracture resistance of endodontically treated teeth, according to eight investigations [27–32, 34] providing substantial evidence. Ribbond was used in nine investigations [20–25, 30, 32, 34]. Three research [30, 32, 34] comparing Ribbond to EverXposterior indicated that EverXposterior had higher fracture resistance values than Ribbond, whereas one study [25] found the opposite, that Ribbond had higher fracture resistance. Ribbond increases the fracture strength compared to traditional hybrid composites and Vectris, according to three studies [22–24]. When fracture resistance Ribbond was compared to other fiber composites in endodontically treated teeth, it revealed inconsistent data. Interlig fibers were used in two experiments [21, 31]. According to one study [31], EverXposterior possessed higher fracture resistance values than Interlig, while composite impregnated with Interlig owned higher fracture strength values than Ribbond fiber composites, showing limited evidence.

## Discussion

This is the first systematic review to evaluate the fracture resistance of fiber-reinforced composites in endodontically treated teeth. This review was con-ducted to assess the resistance to fracture of fiber-reinforced composites when compared to other core restorations following endodontic therapy. The fracture resistance of endodontically treated teeth was lower than that of untreated teeth. Endodontic therapy weakens the tooth structure and makes it more fracture-prone [92, 93]. When compared to traditional hybrid composites, fiber-reinforced compo-sites were able to recover the lost fracture resistance of teeth following root canal treatment in the majority of studies examined.

Discussing the failure mechanisms, brittle failure is caused by fiber-matrix adhesion. The increased matrix damage caused by a combination of the increased test speed and the interfacial bond strength can be seen, as the bunch fiber pull-out [93, 94]. This bunch fiber pull-out indicates that the fiber-matrix interfacial bond strength was exceeded before the composite’s tensile failure strength was reached at this loading rate. As a result of the increased strength of the glass fibers, the observed rate dependency of failure strength is explained. Also, increased test speed increases fiber tensile strength and modulus, allowing the fiber-matrix interfacial bond to be exceeded before the composite’s tensile failure strength [93, 94]. Matrix debonding happens as these fibers are pushed out, resulting in matrix cracking and disintegration [93, 94]. Because of the monoblock effect, fibres have the ability to alter the stress. This in turn aids in distributing stress throughout the tooth’s long axis [95]. Additionally, it can inhibit the formation of crack, as a result of stress transfer from the polymer matrix to the fibres [95, 96]. As core materials, fibres including polyethylene, glass, and short fiber-reinforced composites have been employed. Composite materials reinforced with polyethylene fibres aid in modifying the pat-tern of stresses as well as their distribution and transfer. Glass fibres are sufficiently aesthetically pleasing and have a strengthening capacity [97]. EverX posterior has multidirectional, discontinuous fibres that operate as a dentin substitute, boost strength, and increase the load-bearing capability of the material.

The utilization of high aspect ratio microstructural filler units and orienting these fillers away from the propagation of fracture were key strategies for strengthening this dental material [42, 43]. A Bis-GMA, PMMA, and TEGDMA semi-interpenetrating matrix network, as well as short E-glass fibers and barium glass, make up the Short Fiber-reinforced Composite (SFRC) substructure. It was designed to mimic the fibrous structure of dentin, which makes up around 75 per-cent of the total filler fraction and has a high aspect particle/fiber ratio [98, 99]. Filler loading in this manner improves mechanical qualities such as flexural strength and fracture resistance [99, 100]. Crack-deflecting or crack-bridging processes assist modify stress dynamics when the length of the fiber is more than the critical length of the fiber that is 0.5–1.6 mm [101]. Fracture occurs when interparticle cracks form as it passes through the resin matrix. The filler particles which are linear aid in fracture deflection by diverting the crack away from the region where there is high stress. The simple crack bending lowers the stress distribution and helps joining the particle and allowing the bridge to form. This bridge toughening phenomenon is caused by the twisting of reinforced fibers, which aids in elastic spanning as well as friction between the fibers and their enclosing through the debonding process known as frictional bridging [46]. The crack bridging zone does not degrade at the crack tale due to the high fracture toughness of Ever-X Posterior, which is around 2.4 Mpam^1/2^, increasing the crack resistance curve (R-Curve) [102]. These composites’ anisotropic polymerization contraction behavior helps to reduce shrinkage stress. The plastic IPN matrix is projected to absorb the residual contraction stresses during polymerization, closing the gap between the tooth-restoration continuum [98, 102].

The ability of a material to resist crack propagation under functional stresses is known as fracture toughness. The matrix-filler interface bonding, regardless of the filler form or size, is a significant element that determines fracture toughness. An important parameter to assess a material’s ability to withstand fracture is fracture toughness. It determines the amount of energy needed for a material to fracture and spread to the point of catastrophic failure. Since stresses that a material would typically accept build towards the defective edge in this case, the level of stress needed to trigger a fracture would typically be lower the larger the defect. The ability to fracture is a good indicator of the clinical performance of composite restorations. A material with a high rate of fracture toughness is less prone to chipping or fracture.

Ribbond has strong microtensile bond strength; therefore, employing it on the tensile side of a composite repair will improve its flexural qualities [9]. 215 fibers make up polyethylene longitudinal filaments. These fibers soak up and distribute stress on the tooth structure, reducing stress. The durability, stability, and shear strength of this polyethylene fiber are improved by a unique pattern of cross-linked locking stitched threads [9, 23]. Ribboned polyethylene fiber placed in the flowable composite bed helps to maintain the tooth by raising the elastic modulus and reducing fracture during a composite restoration. Fiber-reinforced composites might outperform traditional hybrid composites in endodontically treated teeth due to their increased physical qualities.

The modulating influence of these fibers on the interfacial tensions generated along the cavity walls through these multidirectional yarns and locked interlaced series of small stitches creating a myriad of load channels might be attributed to reinforcing potential of ribbond [8, 9]. This, in turn, aids in the dispersion of occlusal stresses across a larger area of the restorative material, avoiding rapid fracture formation. Fracture initiation occurs at the fiber particle - resin interface, which is caused primarily by the development of inter-particle cracks inside the resin matrix. Because they are engaged in the crack-blunting mechanism, the insertion of these high flexural modulus short fibers (15.2 Gpa) in this matrix decreases the stress intensity at the fracture tip [9].

The flexural strength and flexural modulus values are highly correlated with the filler volume percentage. The fibers’ involvement in making the material stiffer and more resistant to bending forces both during testing and possibly during use [100]. Previous studies on the assessment of flexural modulus have shown that fibers in composite were able to withstand flexure even at greater load relative to greater sample thickness, but with more deformation before final failure, due to less matrix polymerization and the resulting lack of rigidity. These findings, if validated by more research, would shed more light on a crucial issue pertaining to the amount of deformation and distortion of the material caused by the reduced stiffness, particularly in the interface region.

While comparing inlays and fiber-reinforced composites, moderate evidence was found for fracture resistance of inlays than fiber-reinforced composites in endodontically treated teeth while no evidence could be found for other indirect restorations like crowns or endocrowns. Fracture resistance of inlays and fiber posts using fiber-reinforced composite as core material was found to be moderate, while fracture resistance of bulk fill composites in endodontically treated teeth was found to be contradictory. Surefil SDR, a bulk fill composite, had greater fracture resistance values than SFRC [26, 33]. A polymerization modulator in SureFil SDR Flow decreases the stress that occurs during light polymerization [103, 104]. SDR was shown to have lower polymerization stress and cuspal flexure than other typical flowable composites and was comparable to low shrinkage resin composites [105]. SureFil SDR Flow’s favorable results could be due to its attributes of reduced flexural modulus and slower contraction rate. The evidence was moderate, however fiber-reinforced composites exhibited stronger fracture resistance values than amalgam utilized for core material in MOD cavities of endodontically treated teeth.

Fiber-reinforced composites have higher fracture strength than silorane composites, according to the evidence. In comparison to the nanohybrid group, Silorane is a microhybrid composite with a greater size and lower percentage of filler particles, resulting in early crack propagation and poor fracture resistance.

The disparities in outcomes could be attributed to differences in study designs among the studies examined. The majority of studies found that fiber-reinforced composites restored samples had favorable fractures that were above the CEJ level and could be repaired if desired. However, samples repaired using fiber-reinforced composites cracked unfavorably in research by [29]. This study has a high risk of bias because no periodontal ligament simulation was done. The use of periodontal ligament simulation to assess fracture resistance is controversial. Periodontal ligament and bone must be included in these types of testing, according to Rees and others’ finite element analyses. This reduces the occlusal forces acting on the samples [29].

The artificial periodontal ligament may influence fracture modes, also, the simulation of periodontal ligament has an impact on resistance to fracture [106]. Simulating the periodontal ligament, on the other hand, showed no effect on fracture resistance [107]. According to a study by [108], the microstructure of root dentin changes with age, lowering strength and fatigue resistance. Near the apex, the most severe degradation was discovered, which contributed to the occurrence of vertical root fracture. The final apical diameter differed amongst the studies included in this analysis. Previous studies [109, 110] demonstrated that different canal tapering preparation procedures using various files systems in rotary motion decreased the fracture resistance.

According to [111], increasing apical expansion or canal taper did not enhance the probability of root fracture. Endodontically treated teeth’s fracture susceptibility may be affected by the subsequent irrigation treatment [112, 113]. NaOCl solution was used as the final irrigation in the majority of the clinical trials. On the other side, the concentration, amount, and application time of these solutions varied between investigations. Root canal dentin microhardness was reduced by these irrigant solutions, which could lead to vertical root fracture [114, 115]. The plunger tip diameter, position, and speed varied between experiments. Future research should look into the impact of these variables on fracture resistance testing. Only two studies [20, 24] used cyclic loading prior to fracture resistance testing. In vitro studies measuring the fracture resistance of composite material benefit from cyclic loading of samples because it mimics the dynamic masticatory loads on the restorative material in the oral cavity.

Blinding was not used in any of the trials in this review. Sample size computation and the clinical steps were not completed by a single clinician in the majority of trials. This raised the possibility of bias. According to the current review’s quality evaluation, the majority of the included studies exhibited a moderate or high ROB. As a consequence, the review’s findings should be treated with caution. Although randomized controlled trials produce the most precise and reliable results, well-designed in vitro research with high methodological quality could help solve clinical difficulties [107]. Systematic reviews of in vitro studies can also aid in the area for future research by recognizing the need for more investigation and re-solving the limitations of previous studies.

By combining different results, meta-analysis is a valuable technique for accumulating and summarizing knowledge in a research field and identifying the overall measure of a treatment’s effect [116]. The listed studies were compared in order to construct a meta-analysis in this study. A meta-analysis was not possible due to the heterogeneity of the included studies. Even modest breaches of some meta-analysis guidelines can result in incorrect conclusions [116]. The meta-analysis may be inaccurate due to differences in root canal treatment processes (thermocycling, cyclic fatigue limit, type and concentration of disinfecting agent, canal preparation and obturation method, and so on) among the included research.

To eliminate inter-operator variability in further in vitro fracture resistance research, it is recommended that each process, including canal preparation, disinfection, obturation, cavity preparation, and restoration, be conducted by a single operator. Also included are thermocycling, cyclic loading, and periodontal ligament simulation, as well as following the manufacturer’s guidelines for preparation and restoration. The plunger tip’s diameter, orientation, and pace may have an impact on the end output. As a result, some test requirements should be addressed when designing these studies. With such standardizations, the quality and transparency of in vitro fracture resistance investigations of endodontically treated teeth will increase.

### Implications for research

Well-designed randomized controlled trials should be done to provide evidence-based principles for clinical practice.

## Conclusion

Within the scope of this review, research suggests that using fiber-reinforced composites in endodontically treated teeth might increase fracture resistance compared to traditional hybrid composites although the quality of evidence of included studies was low. This review strongly suggests the development of well-designed randomized clinical trials to test the clinical performance of fiber-reinforced composites compared to other core restorations in endodontically treated teeth.

## Data Availability

The data will be available on reasonable request from the corresponding author.
